# Decreased Serum Brain-Derived Neurotrophic Factor (BDNF) Levels in Patients with Alzheimer’s Disease (AD): A Systematic Review and Meta-Analysis

**DOI:** 10.3390/ijms20020257

**Published:** 2019-01-10

**Authors:** Ted Kheng Siang Ng, Cyrus Su Hui Ho, Wilson Wai San Tam, Ee Heok Kua, Roger Chun-Man Ho

**Affiliations:** 1 Department of Psychological Medicine, Yong Loo Lin School of Medicine, National University of Singapore, Singapore 119228, Singapore; ted.ng@duke-nus.edu.sg (T.K.S.N.); su_hui_ho@nuhs.edu.sg (C.S.H.H.); pcmkeh@nus.edu.sg (E.H.K.); 2 Department of Psychological Medicine, National University Hospital, Singapore 119074, Singapore; 3 Alice Lee School of Nursing, Yong Loo Lin School of Medicine, National University of Singapore, Singapore 119228, Singapore; nurtwsw@nus.edu.sg; 4 Biomedical Global Institute of Healthcare Research & Technology (BIGHEART), National University of Singapore, Singapore 119228, Singapore; pcmrhcm@nus.edu.sg; 5 Center of Excellence in Behavioral Medicine, Nguyen Tat Thanh University, Ho Chi Minh City 70000, Vietnam; pcmrhcm@nus.edu.sg; 6 Faculty of Education, Huaibei Normal University, 100 Dongshan Road, Huaibei 235000, Anhui, China

**Keywords:** systematic review, meta-analysis, meta-regression, BDNF, Alzheimer’s disease, mild cognitive impairment, cognition

## Abstract

Findings from previous studies reporting the levels of serum brain-derived neurotrophic factor (BDNF) in patients with Alzheimer’s disease (AD) and individuals with mild cognitive impairment (MCI) have been conflicting. Hence, we performed a meta-analysis to examine the aggregate levels of serum BDNF in patients with AD and individuals with MCI, in comparison with healthy controls. Fifteen studies were included for the comparison between AD and healthy control (HC) (*n* = 2067). Serum BDNF levels were significantly lower in patients with AD (SMD: −0.282; 95% confidence interval [CI]: −0.535 to −0.028; significant heterogeneity: I^2^ = 83.962). Meta-regression identified age (*p* < 0.001) and MMSE scores (*p* < 0.001) to be the significant moderators that could explain the heterogeneity in findings in these studies. Additionally, there were no significant differences in serum BDNF levels between patients with AD and MCI (eight studies, *n* = 906) and between MCI and HC (nine studies, *n* = 5090). In all, patients with AD, but not MCI, have significantly lower serum BDNF levels compared to healthy controls. This meta-analysis confirmed the direction of change in serum BDNF levels in dementia. This finding suggests that a significant change in peripheral BDNF levels can only be detected at the late stage of the dementia spectrum. Molecular mechanisms, implications on interventional trials, and future directions for studies examining BDNF in dementia were discussed.

## 1. Introduction

Alzheimer’s disease (AD) is the sixth leading cause of death in the United States and is the most prevalent dementia worldwide, with approximately 50–60% of all the dementia cases amongst elderly over 65 years old attributed to AD [1,2,3]. Globally, there are approximately 36 million people suffering from AD or other types of dementia, and the number of patients with dementia was predicted to reach 80 million by 2050 worldwide [4].

In further understanding of this debilitating disease, there have been numerous neurochemical changes identified in patients suffering from AD. The pathological changes include the aggregation of amyloid β-42, increased phosphorylated tau leading to the formation of neurofibrillary tangles [5], heightened low-grade inflammation or inflammaging, reduction in cholinergic function [6], and changes in neurotrophic factors such as brain-derived neurotropic factor (BDNF) levels.

BDNF plays a prominent role in modulating cognition and memory. BDNF is a neurotrophin that belongs to a family of proteins that promote the survival, functions, and development of neurons [7]. The expression of the *BDNF* gene can be found in the cortex, hippocampus, and basal forebrain regions that are indispensable for memory, learning, and higher cognitive function. BDNF enhances neurogenesis and neurotransmission across the synapses, promotes synaptic growth, and modulates synaptic plasticity [8]. BDNF also induces hippocampal long-term potentiation, which is important for memory formation [8]. Weinstein et al. found that higher peripheral BDNF levels protect the older adults against AD. By having BDNF levels higher by one standard deviation, the risk for AD or dementia was lowered by 33% [9].

For clinical studies involving patients with AD, the investigation of the peripheral levels of BDNF remains a controversial topic. While there have been various studies that reported higher peripheral BDNF levels in patients with AD when compared to healthy controls, there were studies that reported results in the opposite direction. Furthermore, there was one study that reported no difference in the levels of BDNF between patients with AD and healthy controls (HC) [10]. Conflicting findings are also present in the literature examining serum BDNF between mild cognitive impairment (MCI) and HC [3,11,12,13,14,15,16,17]. A large study with *n* = 4463 demonstrated a borderline association of serum BDNF levels with MCI (odds ratio, 95% confidence interval: 1.41, 1.00–1.99). However, the confidence interval included the value of one, which made the interpretation difficult [18]. Amongst the potential causes for these discrepancies in findings may include differences in the recruitment process, diagnostic criteria, stages of the diseases, age, mini-mental state examination (MMSE) scores, sex, and education [3]. Another important moderator in these measurements is whether the pro or mature form of BDNF was measured. The consideration of this issue is imperative, although often neglected, as the two forms have opposing effects, in that pro-BDNF promotes cell death, while mature BNF has the opposite function by promoting cell survival [2]. Due to the inconclusive findings to date, a systematic review and meta-analysis is warranted to examine the serum levels of BDNF in patients suffering from AD and individuals with MCI, in comparison with healthy controls.

We examined the aggregate levels of serum BDNF from three groups of subjects representing different stages at the spectrum of neurocognitive disorders, gathered from cross-sectional studies. We proposed two hypotheses in this study. First, we hypothesized that patients with AD have lower serum levels of BDNF as compared to healthy controls and individuals with MCI. Secondly, individuals with MCI have lower serum levels of BDNF as compared to healthy controls. 

## 2. Methods

A comprehensive literature search was performed from the inception of the study through 2017 on these online databases: Pubmed, Embase, PsychINFO, BIOSIS, Science Direct, and Cochrane CENTRAL. The search terms that were used were “BDNF”, “Brain-derived neurotrophic factor”, “mild cognitive impairment”, and “Alzheimer’s Disease”. The search was limited to English articles only. 

### 2.1. Inclusion Criteria

We sought all of the cross-sectional and case-control studies that examined the mean or median serum level of BDNF as the main variable of interest. The studies were included if they (1) were cross-sectional or case-control studies; (2) analyzed the serum levels of BDNF as one of the main variables of interest; (3) compared the serum levels of BDNF between two groups of research participants, which are between either subjects with MCI or AD and a healthy controls group; and (4) compared the serum levels of BDNF for all three groups of subjects, AD, MCI, and healthy control.

### 2.2. Exclusion Criteria

**Figure 1:**
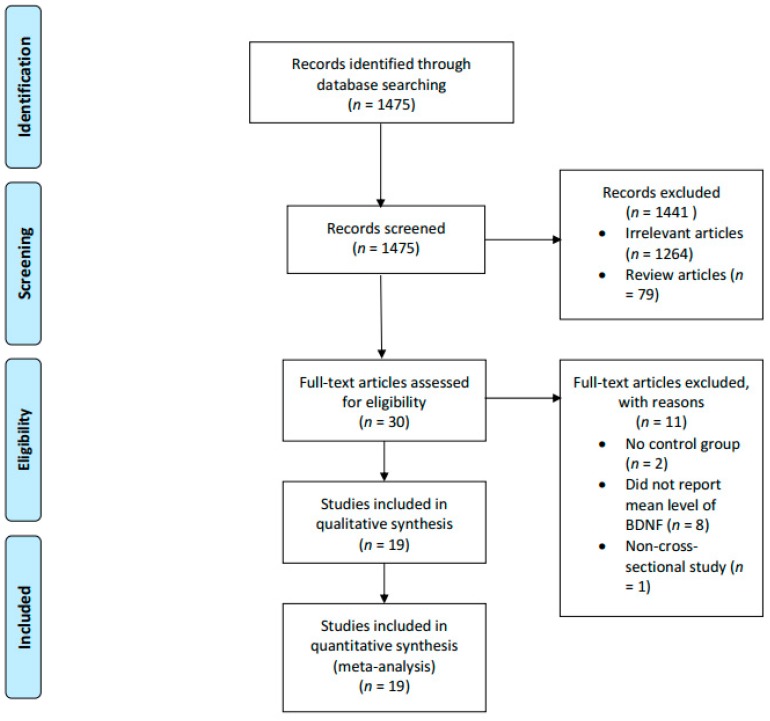
PRISMA diagram describing the process of study selection.

Studies were excluded if the topics were unrelated to BDNF, MCI, or AD, or if they were review articles or animal studies. Secondly, studies that reported only the levels of BDNF for participants with MCI or AD without comparing to a healthy control group were also excluded. Thirdly, studies that did not report the mean nor median levels of serum BDNF levels were also excluded. In the end, we analyzed 19 studies that fulfilled both the inclusion and exclusion criteria (Figure 1).

## 3. Statistical Analyses

Comprehensive Meta-Analysis Version 2.0 (New Jersey, NJ, USA) was used to perform all of the statistical analyses. For certain studies with only median and interquartile ranges available in the manuscripts, estimations of the means and standard deviations were performed according to Hozo et al. [19]. The random effects model was used for meta-analysis to account for potential heterogeneity in the studies that were reviewed, and forest plots were subsequently generated. Standardized mean differences (SMD) were calculated and represented the differences in the mean BDNF levels between the patients with AD, individuals with MCI, and healthy controls. Confidence intervals of 95% were also included. Tests of heterogeneity were conducted with the Q-statistic that is distributed as a Chi-square distribution under the assumption of homogeneity. Heterogeneity is the systematic difference between the results of the studies that cannot be attributed simply to chance. The I^2^ statistic was utilized to examine the between-study heterogeneity. As a guide, I^2^ = 0% suggests no heterogeneity, I^2^ = 25% suggests low heterogeneity, I^2^ = 50% suggests moderate heterogeneity, and I^2^ = 75% suggests high heterogeneity [20]. For examining publication bias, Egger’s regression tests were performed.

For any models with high heterogeneity, meta-regression was performed to identify the sources of heterogeneity, which might have contributed to the heterogeneity in serum BDNF levels in various studies that were included. In the meta-regression analysis, regression coefficients (β) and the associated z and *p*-values were reported.

## 4. Results

From an initial 1475 potentially relevant studies, 1441 studies were excluded after the initial screening. The remaining 30 potential studies were screened against the inclusion and exclusion criteria. Eleven studies were further excluded, and in the end, 19 studies were included in our analyses. 

Fifteen out of the 19 studies provided sufficient data to allow for the comparison of the serum BDNF levels between AD and healthy control groups (Table 1). Eight studies included sufficient data for the comparison of serum BDNF levels between AD and MCI groups. Nine of the 19 studies reported mean/median serum levels of MCI and healthy control groups for comparison. The majority of the studies only reported four main moderators of BDNF: namely age, years of education, mini-mental state examination (MMSE) scores, and sex. Hence, no other confounders or moderators were included in the analyses, although they are known to affect peripheral BDNF levels.

In addition, Table 2 summarizes the key methodological properties and quality of the 19 eligible studies. Notably, most of the studies did not clearly describe the study design nor performance power and sample size calculation. Only one study reported power and sample size calculation [11]. The years since AD/MCI diagnosis on enrollment into the studies and disease durations were also not reported in all of the studies. Approximately half of the reviewed studies reported psychotropic medication usage, covering a wide range of medications. For laboratory measurements, the masking of laboratory staffs measuring serum brain-derived neurotrophic factor (BDNF) levels was also not reported in all of the studies. Although all of the studies reported the assays that were used to measure serum BDNF, assays from a few different manufacturers were employed.

## 5. Forrest Plots

**Figure 2:**
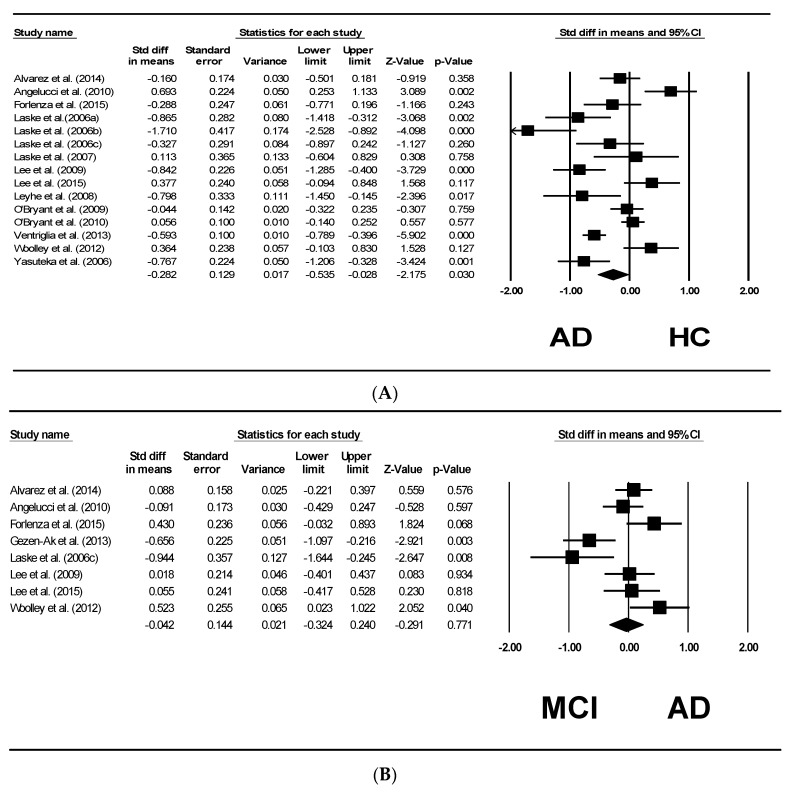

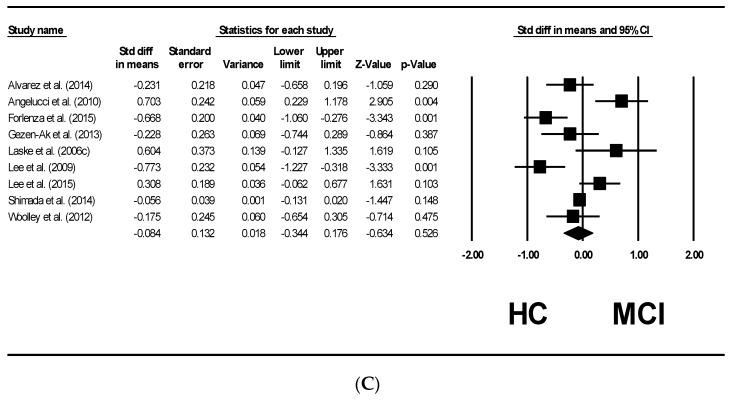
Meta-analysis of serum BDNF levels between AD, MCI, and healthy controls (HC). (**A**) Forest plots depicting the standardized mean differences of serum BDNF levels of patients with AD compared to healthy controls; (**B**) Forest plots depicting the standardized mean differences of serum BDNF levels of individuals with MCI compared to AD; (**C**) Forest plots depicting the standardized mean differences of serum BDNF levels of individuals with healthy controls compared to MCI.

Figure 2A shows the results of the 15 studies [3,11,12,13,14,16,17,21,22,23,24,25,26,27,28] comparing the serum BDNF levels between the patients with AD and healthy controls. From this plot, patients with AD were significantly lower in serum BDNF levels compared to healthy controls (pooled SMD with random effects model: −0.282, with 95% CI: −0.535 to −0.028, *z* = −2.175, *p =* 0.030). Between-study heterogeneity was found to be significant (*τ*^2^ = 0.193, *Q* = 87.294, df = 14, *p <* 0.001, I^2^ = 83.962). After undertaking meta-regression analysis to further explore the effects of the four reported sources of heterogeneity, we found that the mean age contributed significantly to the heterogeneity. (β = 0.0548, *z* = 3.32, *p <* 0.001). MMSE scores had significant effects on the heterogeneity as well (β = 0.0518, z = 3.52, *p <* 0.001). The other two moderators, sex/percentage of female (*p =* 0.1764) and years of education (*p =* 0.2909), have no effects on the heterogeneity of the studies included (Table 3).

Figure 2B shows eight studies [3,11,12,13,14,15,16,17,29] that compared the serum BDNF levels between patients with AD and individuals with MCI. It was found that there were no significant differences in the serum BDNF levels between the two groups (pooled SMD with random effects model: −0.042, 95% CI: −0.324 to 0.240, *z* = −0.291, *p =* 0.771).

Figure 2C shows nine studies [3,11,12,13,14,16,17,18,29] comparing the serum BDNF levels between individuals with MCI and healthy controls. No significant difference was observed (pooled SMD with random effects model: −0.084, 95% CI= −0.344 to 0.176, z = −0.634, *p =* 0.526).

## 6. Publication Bias

Publication bias was tested using Egger’s regression test. Based on the test, there was no publication bias in the serum BDNF levels between the patients with AD and healthy controls (intercept = −0.78, *p =* 0.6278). Publication bias was also not evident in the comparison between other groups.

## 7. Discussions

This study compared the serum levels of BDNF in patients with AD, individuals with MCI, and healthy controls. We concluded that the BDNF levels in the serum of AD patients were significantly lower than healthy controls (15 studies, *n* = 2067). There were no statistically significant differences in serum BDNF levels between patients with AD and individuals with MCI (eight studies, *n* = 906) and between individuals with MCI and healthy controls (nine studies, *n* = 5090), although there were slightly reduced levels in both the comparisons. Meta-regression identified age (*p <* 0.001) and MMSE scores (*p <* 0.001) to be the significant moderators that could explain the heterogeneity in findings in these studies.

We postulate that the decline in serum BDNF levels is a late-stage event in the disease trajectory of AD. Hence, the changes in serum BDNF levels cannot be detected significantly in individuals with MCI yet. There were also substantial reports suggesting that there may be an early stage in the trajectory of the condition where BDNF levels increased as a neuroprotection strategy in response to various insults [14], which was supported by a few of the studies included here reporting increased serum BDNF levels in individuals with MCI. On the other hand, BDNF levels in individuals with MCI may be dependent on various biological properties, lifestyles, and psychosocial factors interacting in an intricate way. Hence, there may be individuals with MCI lacking the protective factors and strategies resulting in the decreased serum BDNF levels. This may have resulted in the differences of the mean serum BDNF levels in individuals with MCI, as reported by the studies covered in this review, with some reporting elevated levels, while other studies found decreased levels (Table 1, Figure 2B,C). However, for patients with AD, serum BDNF levels can be detected as significantly decreased (Figure 2A). The reason might be that the protective and other factors were not able to sustain BDNF levels when neuronal damages were extensive in AD. In patients with AD, cognitive reserves may have largely exhausted, neuronal damages were beyond repairs by BDNF, and the compensatory mechanism failed, resulting in the statistically significant decrease in the serum BDNF levels in patients with AD when compared to healthy controls (Figure 2A).

Meta-regression identified age (*p <* 0.001) and MMSE scores (*p <* 0.001) to be the significant moderators that could explain the heterogeneity in and possibly contradictory findings in these studies (Table 2). We found that the older the subjects and the higher the MMSE scores, there were greater standard differences in the means of serum BDNF levels. This might be one of the reasons why for MCI, which has higher MMSE scores, no significant difference in serum BDNF levels has been detected yet. Further research is warranted to explore the effects of age and MMSE scores on serum BDNF levels. Although they are other known moderators that could have contributed to the heterogeneity in the peripheral BDNF levels in these studies, they are not regularly reported. Thus, we are unable to examine the effects of these other moderators.

One such moderator is psychotropic medication usage. However, only approximately half of the studies reviewed here reported this moderator. Psychotropic medications, especially for treating cognitive disorder and major depressive disorder (MDD), have been shown to affect serum BDNF levels. For example, a study by Leyhe et al. showed that by treating patients with AD with donepezil for 15 months, serum BDNF levels were restored to the levels similar to those of healthy controls [23]. Furthermore, antidepressant-naïve patients with MDD that have been treated with antidepressants have their serum BDNF levels recovered to basal levels as well [30]. 

Additionally, power and sample size calculation need to be considered, especially in studies with a relatively small sample size, which is typical of biomarker studies, including the majority of the studies reviewed here. A cohort size of 60 has been postulated to be able to detect statistically significant differences in BDNF levels, assuming a 20% difference, with a power of 0.80 and type-I error of 0.05 [31]. If a 10% difference in BDNF levels was hypothesized, a relatively large cohort size of 200 is necessary. In fact, this sample size requirement might have rendered some of the studies underpowered, and hence unable to detect a statistically significant difference [31].

Our meta-regression results suggested that sex was not a significant moderator in the studies reviewed. Furthermore, other studies have shown that serum BDNF levels have no significant difference when stratified by sex [31]. Hence, we propose that sex per se might not have a main and strong effect on serum BDNF levels. However, it was reviewed that there could be an interaction effect of sex with other moderators, such as the *APOE* genotype and medications [32]. It was further proposed that although the influence of sex on the diagnostic utility of BDNF as AD biomarkers is limited, its prognostic utility in a large-scale cohort study should be investigated [32].

Apart from clinical and statistical considerations, the handling and measurement processes of the blood samples in the laboratory are equally important. One important aspect that is often overlooked and underreported in clinical research is the masking of laboratory staffs to the diagnostic statuses of the samples. Unsurprisingly, none of the studies reviewed here reported this variable. Furthermore, a few different serum BDNF measurement assays produced by different manufacturers were employed in the studies that were reviewed. Polacchini et al. reviewed six commercially available assays, and concluded that only two out of the six assays selectively recognize mature BDNF, with the rest of the assays giving readings that combined the signals from both pro-BDNF and mature BDNF [33]. The two assays that have been reported to be specific for mature BDNF were from Aviscera-Bioscience and R&D System-Quantikine. The majority of the studies reviewed here employed the assays from R&D Systems. This issue of antibody-specificity in recognizing only the mature form of BDNF is critical, as serum contains both forms, which have opposing effects. Thus, our recommendation for future studies would be to use those assays that are specific to only the mature form to reduce study heterogeneity in order to facilitate comparisons across studies. In another study, the assay manufactured by Promega-Emax has been identified as the most widely-used BDNF kit, although it requires overnight plate-coating, which would further contribute to study heterogeneity [33]. The species-specificity of the assay to human samples is another important consideration for future studies. Promega-Emax assay did not declare human species-specificity, while the assays produced by Biosensis and Millipore-Chemikine showed cross-reactivity with rodent species [33]. Other considerations in the laboratory include batch effects, intra-assay and inter-assay variation, total processing time, sensitivity, and the range of detection. Taken together, mature BDNF form-specific and human-specific assays are recommended for future studies. Naegelin et al. have shown that BDNF can indeed be reliably measured in human serum [31]. However, based on this review, we identified that there are still a number of issues needing standardization in measurement as discussed above. Only with the standardization of measurement methods would future studies be less heterogeneous and more comparable across studies. This is a critical step in facilitating the establishment of the clinical threshold values of serum BDNF in cognitive disorders, and eventually propelling the use of serum BDNF in the clinics, enabling the translation of biomarker research. 

To our knowledge, this is among the first systematic review and meta-analysis on serum BDNF levels in patients with AD and individuals with MCI, compared with healthy controls. This covered the whole spectrum of the disease trajectory. With the availabilities of the four most common moderators, we managed to perform meta-regression analyses. The analyses identified the important factors that contributed to the observed heterogeneity in serum BDNF levels in the studies reviewed, which necessitates controlling for these factors in future studies. We further reviewed other moderators that could potentially confound serum BDNF findings and provide recommendations for future studies. In addition, this review included an a priori search strategy, a comprehensive literature review, and the incorporation of strict inclusion and exclusion criteria, supporting the robustness of the study. 

There are a number of limitations in this study. As most of the studies reviewed were conducted in the Western countries, there might be differences in the factors involved that we were not able to examine. Amongst them are psychosocial factors, such as culture and ethnicity, and genetic factors, such as the allelic differences in the *BDNF* gene, which has been shown to differentially affect peripheral BDNF levels. This gap of knowledge calls for future investigations of peripheral BDNF levels in the other populations. On this note, only a limited number of moderators was included in the meta-regression due to the scarce data presented by the studies reviewed. Most of the studies did not report moderators other than the ones examined above. Hence, some of the well-established confounders of BDNF levels, such as co-morbidities, psychosocial and lifestyle factors, the use of antidepressants, and *BDNF Val66Met* polymorphism were not able to be taken into account in the meta-regression analysis. Furthermore, most of the studies included in this meta-analysis also did not differentiate early-onset nor late-onset AD, nor the severity of AD, which rendered the differentiation of peripheral BDNF levels in these subgroups infeasible. The differentiation of these subgroups may provide a higher precision of the levels of BDNF in patients with AD. However, we also note that by further segregating the subjects into subgroups, the analytical power of the meta-analysis may be compromised due to the small number of studies in each subgroup.

## 8. Molecular Mechanisms of the Reduced Serum BDNF Levels in AD

There are a few known molecular mechanisms that regulate BDNF levels. Among those are the regulated lysosomal degradation of BDNF proteins [34], regulated proteins expression via the epigenetic regulation of BDNF [35,36], the regulated BDNF release by platelets [37], and the regulated sorting and cleavage of pro-BDNF to mature BDNF [38]. Reviewed below are some of the plausible mechanisms that could have contributed to the decreased serum BDNF levels in patients with AD.

The glucocorticoid (GC) hypothesis of brain aging and Alzheimer’s disease postulates that continuous exposure to glucocorticoids such as corticosterone promotes the aging of the hippocampus and causes AD [39]. A study found that corticosterone decreased *BDNF* expression at both the mRNA and protein levels [40]. A study by Connor et al. reported that the administration of corticosterone decreased BDNF mRNA by as much as 70% [41]. Decreased mRNA levels of BDNF in post-mortem brains have also been shown in several studies [37,38,39].

Another molecular mechanism that contributed to the significantly decreased serum BDNF levels in patients with AD may be modulated by amyloid β-42. A study found that three of the seven human BDNF mRNA transcripts were specifically downregulated in AD. They also found that oligomeric Aβ_1–42_ decreased phosphorylated CAMP responsive element binding protein(CREB) and the major *BDNF* transcripts, transcripts IV and V, in a human neuroblastoma cell line model [42]. Accordingly, a study on AD post-mortem brains revealed significant decreases in total CREB and phospho-CREB levels. These decreased phospho-CREB and CREB levels could be due to the increase in corticosterone levels as well [43]. In all, both glucocorticoid and amyloid β-42 may decrease the mRNA transcripts of BDNF through the regulation of total and phospho-CREB levels, resulting in decreased serum BDNF levels in patients with AD. 

Additionally, platelets may serve as a storage compartment for BDNF and may play an important role in the regulation of BDNF levels in the serum [37]. Correspondingly, Karege et al. reported reduced BDNF release from the platelets in the serum of patients with major depressive disorder [44]. It will be worth investigating whether similar phenomenon happens in patients with AD as well. The epigenetic regulation of BDNF levels through DNA methylation and miRNA regulation [45] may have also contributed to the decreased serum BDNF levels in AD and warrant future clinical studies. On epigenetic regulation of BDNF, Catteneo et al. has performed an excellent and thorough review [46].

## 9. Evidence and Protective Mechanisms of Actions of BDNF

BDNF offers protection against oxidative stress via CREB-mediated transcription [47]. A-CREB, a potent negative regulator of CREB/CRE-mediated transcription, blocked the neuroprotective effects of BDNF and rendered cultured cells vulnerable to excitatory insults [47]. Specifically, in cell culture, BDNF conferred protection against hydrogen peroxide insults, whereas in vivo, BDNF induced CREB expression, and this was associated with a decrease in oxidative load [47]. CREB is a regulator of gene transcription, and the genes that it regulates to confer protection against oxidative stress are yet to be identified via mRNA microarray and proteomics. When challenged by glutamate, an excitocytotoxin, BDNF protected the cultured hippocampal neuron against neuronal insults [48]. On the other hand, the administration of a BDNF antibody completely abolished the excitoprotective action of BDNF [48]. In rat models, when rats infused with a BDNF-blocking antibody were challenged with kainate, an excitocytotoxin, the excitoprotective effect of BDNF was abolished as evidenced by the extensive loss of CA3 neurons [48].

## 10. Interventions to Increase BDNF Levels

Below, some of the psychosocial interventional strategies that had been demonstrated to improve BDNF levels and result in concomitant improvements in cognitive functions are reviewed.

Medications have been shown to have a positive impact on BDNF levels. Correspondingly, the pharmaceutical compounds that are used to alleviate the symptoms of AD are attractive targets, since they have well-characterized therapeutic and side effects. For example, memantine at a clinically-relevant dose markedly increased the mRNA levels of BDNF in the limbic cortex, and this effect was widespread and dose-dependent. The effects of memantine on the mRNA levels of BDNF were also reflected in the changes in the protein levels of BDNF [49]. Another study using macaques also showed that memantine specifically upregulated the mRNA and protein expression of BDNF [50]. For studies involving human subjects, after treating AD patients with donepezil for 15 months, the serum BDNF levels in the patients with AD increased significantly to the level equivalent to those of healthy controls [23]. In all, these evidences suggested the potential of the medications on modifying BDNF levels and improving cognition.

Apart from medications, lifestyles changes were proven to be valuable in increasing BDNF levels. One of the lifestyle changes is through dietary restriction and healthy eating. It was found that in the rat model, dietary restriction induced the gene expression of BDNF, coupled with increased number of newly sprouted neurons [51]. A study by Duan et al. also showed that BDNF levels were significantly increased in the hippocampus, cerebral cortex, and striatum of rats that were fed with dietary restricted and healthy diet [48]. Another study on human participants illustrated the importance of controlling body weight on circulating BDNF levels [52]. Chronic sleep deprivation has also been shown to reduce BDNF levels in the experimental model of rats [53]. Additionally, increased stress levels and sleep loss have been implicated in reducing serum BDNF levels in a clinical study [54]. Taken together, these two studies highlighted the importance of sleep on maintaining BDNF levels.

Another plausible strategy is exercise. Ferris et al. found that serum BDNF levels were significantly increased after exercising, and the higher the intensity of the exercises, the higher the increases in BDNF levels [55]. Additionally, it was shown that consistent daily aerobic exercise over a one-year period resulted in increased serum BDNF levels, increased hippocampal volumes, and improved spatial memory [56]. Laske et al. found that after a short 30-min exercise, there were significant increases in the BDNF levels in patients with major depressive disorder, to the BDNF levels that were similar to those in healthy controls [57]. Lastly, physical therapy intervention (PTI) was also shown to be able to increase BDNF levels in elderly women [58].

In addition, meditation is another potential practice that could increase the levels of BDNF. Meditation is a broad term that encompasses many different practices [59], some of which are transcendental meditation, yoga, and mindfulness practice. Transcendental meditation (TM) involves chanting an individualized mantra with eyes closed for 20 min twice daily. This practice resulted in cortisol levels in the TM practitioners that were three times lower than the controls [60]. This decreased cortisol level might have an impact on the BDNF level. Xiong et al. proposed that by decreasing cortisol levels, the BDNF level may be improved [59]. Meditation practitioners were found to have lower age-related declines in the thicknesses of specific cortical regions [59]. Similarly, yoga has been shown to increase serum BDNF levels in both groups of depressed patients, with and without medication [61].

Other viable methods for increasing BDNF levels and resulted in cognitive improvements that were not discussed above include intellectual stimulation [62], increased consumption of foods and supplements such as curcumin [63], docosahexaenoic acid (DHA) [64], and green teas, which contain polyphenols that were demonstrated being able to increase BDNF levels [65]. Furthermore, there are synergistic effects of exercises and diet, as evidenced by the study of Wu et al., which illustrated that the combined administration of DHA and exercises resulted in increased levels of the activated forms of hippocampal Akt and Ca2+/calmodulin-dependent protein kinase-II (CaMKII), both of which act as downstream signaling mediators of BDNF in an animal model [66].

## 11. Future Directions

The evidence is robust, as evidenced by our analyses, showing that there was a statistically significant difference in the serum BDNF levels in patients with AD when compared to healthy controls. This phenomenon of decreased peripheral BDNF levels suggests another pathological process in AD that warrants greater attention, in light of repeated failure of AD trials targeting mainly amyloid β and tau proteins. Increased effort in clinical research, specifically focusing on longitudinal follow-up studies that are able to delineate the temporal relationship of BDNF in relation to cognitive scores and brain anatomical, functional and metabolic changes using imaging techniques would be particularly valuable in gaining deeper insights into this area. In reference to the limitations of the limited variables reported in the studies reviewed, the various confounders of BDNF levels that were reviewed here need to be examined in these proposed future longitudinal studies. We propose that the examination of these confounders, whether regarding the individual and/or synergistic effects on peripheral BDNF, might be the “holy grail” of the field examining BDNF and cognitive disorders. Emphasis should be placed on the subgroup of patients with comorbid MDD and those taking psychotropic medications. Various demographics, study design, and laboratory parameters as discussed are equally important. With these proposed large and clinically well-defined and well-controlled cohorts, the trajectory of BDNF in relation with cognition in AD and dementia could be delineated. These prospective studies could provide the foundation for the usage of BDNF in combination with other biomarkers in the predictive, diagnostic, and treatment response context-of-use in AD and dementia. Lastly, novel psychosocial interventions that increase the levels of peripheral BDNF levels, which result in concomitant improved cognitive functions, represents another relatively uncharted but very promising avenue.

## 12. Conclusions

There have been conflicting data on peripheral BDNF levels in the different stages of AD trajectory. Therefore, elucidating the direction of changes in peripheral BDNF levels in the spectrum of cognitive impairment from HC to MCI to AD is of dire importance. This study provided pilot data which concluded that there was indeed decreased BDNF in the serum of patients with AD. However, we observed no significant difference in the serum BDNF levels in AD and MCI and in MCI and healthy controls. Differences in age and MMSE scores contributed to the heterogeneity in the findings in the studies that were included. One of the implications of this study is that decreased peripheral BDNF levels in AD could serve as a baseline for future trials to examine the effectiveness of the trials in improving peripheral BDNF levels and observe the concomitant improvements in cognitive domains.

## Figures and Tables

**Table:** (**A**)

Study Authors and Years of Publications	Locations of the Studies	Patients with ad Sites of Recruitment	Case Definitions	AD Serum BDNF Mean (SD)	Numbers of Patients with AD; Ratio Male/Female	Average Age (SD)	Healthy Controls Sites of Recruitment	Methods of Cognitive Assessments	Control Serum BDNF Mean (SD)	Number of Healthy Controls; Ratio Male/Female	Average Age	Total Numbers of Subjects; Ratio Male/Female
**Alvarez et al. (2014) [12]**	A Coruña, Granada and Málaga, Spain	Three institutions specialized in cognitive disorders	DSM-IV and NINCDS-ADRDA criteria	15.16 (9.48)	252; M = 56, F = 196	74.99 (7.35)	Three institutions specialized in cognitive disorders	MMSE and ADAS-cog+	16.73 (11.83)	38; M = 8, F = 30	72.74 (5.69)	290; M = 64, F = 226
**Angelucci et al. (2010) [3]**	Rome, Italy	NA	NINCDS-ADRDA criteria and MMSE	6.07 (1.27)	89 Mild AD: 54; M = 19, F = 35 Moderate-severe AD: 35; M = 8, F = 22	Mild AD (*n* = 54) 74.31 (6.97) Moderate-severe AD (*n* = 35) 77.42 (8.27)	NA	MMSE score >24 and not satisfying the NINCDS-ADRDA criteria for the diagnosis of AD or the MCI Petersen criteria, confirmed by the memory tests of the MDB	5.17 (1.39)	27; M = 10, F = 17	68.48 (6.12)	116; M = 64, F = 54
**Forlenza et al. (2015) [13]**	São Paulo, Brazil	Community-dwelling elders recruited from an ongoing cohort study	NINCDS-ADRDA criteria	0.66 (0.49)	26; M = 8, F = 18	76.8 (6.6)	Community-dwelling elders recruited from an ongoing cohort study	Cambridge Cognitive Test, MMSE and neuropsychological tests	0.84 (0.69)	46; M = 9, F = 37	68.8 (6.8)	72; M = 17, F = 55
**Laske et al. (2007) [21]**	Tübingen, Germany	Outpatients from memory clinic	DSM-V, ICD-10, NINCDS-ADRDA criteria and MMSE	18.60 (2.67)	27; M = 10, F = 17	70.2 (8.6)	Healthy elderly volunteers	Without any organic brain disorders and had to reach MMSE score >27	21.63 (4.70)	28; M = 9, F = 19	70.6 (7.1)	55; M = 19, F = 36
**Laske et al. (2006A) [22]**	Tübingen, Germany	Outpatients from memory clinic	DSM-V, ICD-10, NINCDS-ADRDA criteria and MMSE	18.30 (2.00)	28; M = 11, F = 17	70.4 (NA)	Healthy elderly volunteers	Normal clinical and cognitive status according to clinical examination and MMSE score	21.60 (3.96)	10; M = 6, F = 4	69.1 (NA)	38
**Laske et al. (2006B) [14]**	Tübingen, Germany	Outpatients from memory clinic	DSM-V, ICD-10, NINCDS-ADRDA criteria and MMSE	21.20 (4.18)	30; M = 9, F = 21	71.7 (7.0)	Patients who underwent lumbar puncture for orthopedic or neurologic diagnostic purposes	Have normal CSF cell counts and total protein levels, absence of signs of blood–brain barrier dysfunction or cerebral immunoglobulin G (IgG) synthesis, no cerebral disorders	20.70 (5.20)	10, M = 2, F = 8	70.3 (5.4)	40; M = 11, F = 29
**Laske et al. (2006C) [15]**	Tübingen, Germany	NA	MMSE <26 and ≥21	19.60 (4.50)	30; NA	NA	NA	NA	21.10 (4.70)	20; NA	NA	50; NA
**Lee et al. (2009) [16]**	Busan, South Korea	Patients of the Busan Paik Hospital	MMSE-KC, CERAD-K, CDRS, DSM-IV, NINCDS-ADRDA criteria	22.90 (5.00)	47; M = 14, F = 33	75.1 (6.4)	Patients of the Busan Paik Hospital	MMSE-KC scores >25	27.90 (6.90)	39; M = 16, F = 23	72.8 (5.0)	86; M = 30, F = 56
**Lee et al. (2015) [17]**	Busan, South Korea	Elderly individuals over 60 years of age	MMSE-KC, CERAD-K, CDRS, DSM-IV	29.10 (7.70)	25; NA	73.3 (6.8)	Elderly individuals over 60 years of age	Score >1.5 standard deviations (SD) above the mean of normalized MMSE-KC score	26.80 (5.30)	59; NA	72.0 (5.8)	84; NA
**Leyhe et al. (2008) [23]**	Tübingen, Germany	NA	DSM-IV, ICD-10, NINCDS-ADRDA criteria, MMSE EEG, CT, or MRI were also performed to validate the diagnosis of AD	19.20 (3.70)	19; M = 4, F = 15	70.9 (8.7)	NA	Without any organic brain disorders and MMSE score ≥27	23.20 (6.00)	20; M = 13, F = 7	69.6 (11.6)	39; M = 17, F = 22
**O’Bryant et al. (2009) [24]**	Texas, USA	Participants from TARC	NINCDS-ADRDA criteria	23.50 (7.40)	99; M = 43, F = 56	77.8 (8.17)	Participants from TARC	Normal limits on psychometric assessment and were assigned a CDR global score of 0	23.80 (6.30)	99; M = 39, F = 60	72.01 (8.56)	198; M = 82, F = 116
**O’Bryant et al. (2010) [25]**	Texas, USA	Participants from TARC	NINCDS-ADRDA criteria	31.46 (9.10)	198; M = 68, F = 130	76.63 (8.33)	Participants from TARC	Judged to be within normal limits on consensus review	30.96 (8.82)	201; M = 64, F = 137	70.4 (8.86)	399; M = 132, F = 267
**Platenik et al. (2014) [26]**	Prague, Czech Republic	Department of Psychiatry of the First Faculty of Medicine and General University Hospital	age >50 years; NINCDS-ADRDA Alzheimer’s criteria; brain imaging (magnetic resonance imaging) measuring cortico-subcortical atrophy (atrophy in the hippocampus and temporal corners of the side chambers); no any other organic brain lesions (vascular changes, tumors, intracranial hemorrhage, etc.); MMSE score <26; and no serious unstable somatic disease.	1.72 (NA)	85; M = 34, F = 51	75.6 (7.7)	Department of Psychiatry of the First Faculty of Medicine and General University Hospital	Underwent a psychiatric examination equivalent to that of AD patients, and it was confirmed that they were non-demented, nondepressed, and without any organic brain disorder.	1.68 (NA)	96; M = 30, F = 66	47.8 (16.1)	181; M = 64, F = 117
**Ventriglia et al. (2013) [27]**	Brescia, Italy	Alzheimer’s Unit of a private hospital	NINCDS-ADRDA & DSM-V MMSE for examination of severity	33.16 (12.40)	266; M = 88, F = 178	80.1 (7.1)	Enrolled at the Alzheimer Unit of a private hospital	MMSE scores ≥27/30	39.89 (9.48)	169; M = 83, F = 86	48.0 (15.7)	435; M = 171, F = 264
**Woolley et al. (2012) [11]**	California, USA	Memory and Aging Center	NINCDS-ADRDA criteria	23.00 (11.00)	34; M = 18, F = 16	66.1 (11.3)	Recruited through advertisements in local newspapers & recruitment talks at local senior community centers.	A normal result from neurological examination, a CDR score of 0 & MMSE score ≥28/30.	19.00 (11.00)	38; M = 15, F = 23	69.2 (9.8)	72; M = 33, F = 39
**Yasutake et al. (2006) [28]**	Takatsuki, Japan	NA	NINCDS-ADRDA criteria and CT scan. AD severity was rated according to FAST	14.73 (5.88)	60; M = 20, F = 40	77.93 (7.04)	NA	NA	19.72 (7.53)	33; M = 8, F = 25	71.06 (5.77Z)	93; M = 28, F = 65

**Table:** (**B**)

Study Authors and Years of Publications	Locations of the Studies	Patients with AD Sites of Recruitment	Case Definitions	AD Serum BDNF Mean (SD)	Numbers of Patients with AD; Ratio Male/Female	Average Age	Individuals with MCI Sites of Recruitment	Case Definitions	MCI Serum BDNF Mean (SD)	Numbers of Individuals with MCI; Ratio Male/Female	Average Age	Total Numbers of Subjects; Ratio Male/Female
**Alvarez et al. (2014) [12]**	A Coruña, Granada and Málaga, Spain	Three institutions specialized in cognitive disorders	DSM-IV and NINCDS-ADRDA criteria	15.16 (9.48)	252; M = 56, F = 196	74.99 (7.35)	Subjects evaluated at three institutions specialized in cognitive disorders	Petersen criteria revised	14.33 (9.12)	48; M = 13 F = 35	73.46 (7.57)	300; M = 69, F = 231
**Angelucci et al. (2010) [3]**	Rome, Italy	NA	NINCDS-ADRDA and MMSE	6.07 (1.38)	89 Mild AD: 54; M = 19, F = 35 Moderate-severe AD: 35; M = 8, F = 22	Mild AD (*n* = 54) 74.31 (6.97) Moderate-severe AD (*n* = 35) 77.42 (8.27)	NA	Peterson’s guidelines and MMSE score ≥23	6.20 (1.50)	54; M = 32, F = 27	69.61 (6.65)	143; M = 59, F = 84
**Forlenza et al. (2015) [13]**	São Paulo, Brazil	Community-dwelling elders recruited from an ongoing cohort study	NINCDS-ADRDA criteria	0.66 (0.49)	26; M = 8, F = 18	76.8 (6.6)	Community-dwelling elders recruited from an ongoing cohort study	Mayo Clinic criteria	0.51 (0.27)	62; M = 17, F = 45	72.2 (6.2)	88; M = 25, F = 63
**Gezen-ak et al. (2013) [29]**	Istanbul, Turkey	Behavioral Neurology and Movement Disorder Clinic	DSM-VI and MMSE	0.93 (0.31)	76; NA; EOAD = 22, LOAD = 54	EOAD = 61.1 (4.8); LOAD = 74.22 (3.73)	Behavioral Neurology and Movement Disorder Clinic	NA	1.15 (0.40)	30; NA	74.4 (2.9)	101; NA
**Laske et al. (2006C) [15]**	Tübingen, Germany	NA	MMSE <26 + > or =21	19.60 (4.50)	30; NA	NA	NA	MMSE ≥26	24.10 (5.40)	12; NA	NA	42; NA
**Lee et al. (2009) [16]**	Busan, South Korea	Patients of the Busan Paik Hospital	MMSE-KC, CERAD-K, CDRS, DSM-IV, NINCDS-ADRDA criteria	22.90 (5.00)	47; NA	NA	Patients of the Busan Paik Hospital	CDRS and Peterson’s Criteria	22.80 (6.30)	41; NA	NA	88; NA
**Lee et al. (2015) [17]**	Busan, South Korea	Elderly individuals over 60 years of age	MMSE-KC, CERAD-K, CDRS, DSM-IV	29.10 (7.70)	25; NA	73.3 (6.8)	Elderly individuals over 60 years of age	MMSE-KC, CERAD-K, CDRS, DSM-IV	28.70 (7.00)	55; NA	71.5 (4.7)	80; NA
**Woolley et al. (2012) [11]**	California, USA	Memory and Aging Center	NINCDS-ADRDA criteria	23.00 (11.00)	34; M = 18, F = 16	66.1 (11.3)	Memory and Aging Center	Peterson’s Criteria	17.00 (12.00)	30; M = 17, F = 13	71.3 (11.5)	64; M = 35, F = 29

**Table:** (**C**)

Study Authors and Years of Publications	Locations of the Studies	Individuals with Mci Sites of Recruitment	Case Definitions	MCI Serum BDNF Mean (SD)	Numbers of Individuals with MCI; Ratio Male/Female	Average Age	Healthy Controls Sites of Recruitment	Methods of Cognitive Assessments	Control Serum BDNF Mean (SD)	Numbers of Healthy Controls; Ratio Male/Female	Average Age	Total Numbers of Subjects; Ratio Male/Female
**Alvarez et al. (2014) [12]**	A Coruña, Granada and Málaga, Spain	Three institutions specialized in cognitive disorders	Petersen’s criteria revised	14.33 (9.12)	48; M = 13, F = 35	73.46 (7.57)	Subjects evaluated at three institutions specialized in cognitive disorders	MMSE and ADAS-cog+	16.73 (11.83)	38; M = 8, F = 30	72.74 (5.69)	86; M = 21, F = 65
**Angelucci et al. (2010) [3]**	Rome, Italy	NA	Peterson’s guidelines and MMSE score = or > 23	6.20 (1.50)	54; M = 32, F = 22	69.61 (6.65)	NA	MMSE score >24 and not satisfying the NINCDS-ADRDA criteria nor the Petersen’s criteria, confirmed by the memory tests of the MBD	5.17 (1.39)	27; M = 10, F = 17	68.48 (6.12)	81; M = 42, F = 39
**Forlenza et al. (2015) [13]**	São Paulo, Brazil	Community-dwelling elders recruited from an ongoing cohort study	Mayo Clinic criteria	0.51 (0.27)	62; M = 17, F = 45	72.2 (6.2)	Community-dwelling elders recruited from an ongoing cohort study	Cambridge Cognitive Test, MMSE, and neuropsychological tests	0.84 (0.69)	46; M = 9, F = 37	68.8(6.8)	108; M = 26, F = 82
**Gezen-ak et al. (2013) [29]**	Istanbul, Turkey	Behavioral Neurology and Movement Disorder Clinic	DSM-VI and MMSE	1.15 (0.40)	29; NA	74.4 (2.9)	Behavioral Neurology and Movement Disorder Clinic	NA	1.25 (0.48)	29; NA	72.1 (3.4)	58; NA
**Laske et al. (2006C) [15]**	Tübingen, Germany	NA	MMSE > or = 26	24.10 (5.40)	12; NA	NA	NA	NA	21.10 (4.70)	20; NA	NA	32; NA
**Lee et al. (2009) [16]**	Busan, South Korea	Patients of the Busan Paik Hospital	CDRS and Peterson’s Criteria	22.80 (6.30)	41; M = 17, F = 24	74.1 (5.7)	Patients of the Busan Paik Hospital	MMSE-KC scores >25	27.90 (6.90)	39; M = 16, F = 23	72.8 (5.0)	80; M = 33, F = 47
**Lee et al. (2015) [17]**	Busan, South Korea	Elderly individuals over 60 years of age	MMSE-KC, CERAD-K, CDRS, DSM-IV	28.70 (7.00)	55; NA	71.5 (4.7)	Elderly individuals over 60 years of age	Score >1.5 standard deviations (SD) above the mean MMSE-KC score	26.80 (5.30)	59; NA	72.0 (5.8)	114; NA
**Shimada et al. (2014) [18]**	Obu, Japan	Subjects enrolled in the OSHPE	≤23 points on the MMSE and NCGG-FAT (for screening), Petersen’s Criteria for diagnosis	20.90 (5.30)	827; NA	NA	Enrolled in the OSHPE	NA	21.20 (5.40)	3636; NA	NA	4463; NA
**Woolley et al. (2012) [11]**	California, USA	Memory and Aging Center	Peterson’s Criteria	17.00 (12.00)	30; M = 17, F = 13	71.3 (11.5)	Recruited through advertisements in local newspapers and recruitment talks at local senior community centers.	A normal result from neurological examination, a CDR score of 0, and MMSE score ≥28/30.	19.00 (11.00)	38; M = 15, F = 23	69.2 (9.8)	68; M = 32, F = 36

Abbreviations: DSM-V: The Diagnostic and Statistical Manual of Mental Disorders, Fifth Edition; ICD-10: 10th revision of the International Statistical Classification of Diseases and Related Health Problems; NINCDS-ADRDA: National Institute of Neurological and Communicative Disorders and Stroke (NINCDS) and the Alzheimer’s Disease and Related Disorders Association (ADRDA) criteria; MMSE: Mini-Mental State Examination, ADAS-cog+: The Alzheimer’s Disease Assessment Scale Cognitive Plus; MMSE-KC: Mini-Mental Status Examination: Korean version; CERAD-K: Consortium to Establish a Registry for Alzheimer’s Disease, Korean version; CDR: Clinical Dementia Rating; CDRS: Clinical Dementia Rating Scale; EEG: electroencephalogram; CT: computerized tomography; MRI: magnetic resonance imaging; MDB: Mental Deterioration Battery; TARC: Texas Alzheimer’s Research Consortium; FAST: Functional Assessment Staging; OSHPE: Obu study of health promotion for the elderly; NCGG-FAT: the National Center For Geriatrics And Gerontology-Functional Assessment Tool; USA: United States of America; MBD: Mental Deterioration Battery; NA represents data not reported in the respective studies.

**Table 2:** Key methodological properties and laboratory variables.

Study Authors and Years of Publications	Study Design	Power Calculation and Sample Size Calculation (YES/NO)	Bdnf Lab Technician Performing Elisa Procedure Masked (Yes/No)	Type of BDNF Elisa Assay Kit Used and Manufacturer	Intra-Assay and Inter-Assay Cv (%)	Disease Duration	Psychotropic and Other Medications Usage	Exclusion Criteria; Other Potential Moderators/Confounders Reported
**Alvarez et al. (2014) [12]**	Case-control study	-	-	ELISA kit specific for the quantitative determination of both natural and recombinant human BDNF in cell culture supernatant, serum and plasma (R&D Systems, Inc., Minneapolis, MN, USA) provided by Vitro SA (Spain)	Both <10%	-	SSRIs treatment (yes, no)	Subjects having any other significant neurological or psychiatric disease, active allergies, unstable medical conditions, or clinically significant laboratory abnormalities. Not taking systemic corticosteroids, antiparkinsonian agents, narcotics, or cholinesterase inhibitors for at least one month prior to blood sampling. No clinically significant depression in the medical evaluation and/or scores higher than 15 in the 17-item subscale of the Hamilton Depression Scale. Subjects were not on specific exercise programs. APOE4, apathy (present, absent), dysphoria (present, absent), disease severity (CIBIS+ score), dysphoria, total NPI score and CIBIC+ score
**Angelucci et al. (2010) [3]**	Case-control study	-	-	Sandwich ELISAs (R and D Systems, Minneapolis, MN, USA). This ELISA kit is set in order to measure human mature BDNF.	8,14	-	AChEI or antidepressant drugs; 15.7% of total AD patients were free of treatments at the time of blood collection. 76.5% of AD patients were treated with AChEI and 38% with antidepressants. 14.6% of AD patients, 12.9% of MCI, 11.1% of healthy subjects were prescribed statins at the time of the study.	Exclusion criteria: diabetes, obstructive pulmonary disease or asthma, hematological/oncological disorders, B12 or folate deficiency, pernicious anemia, active gastrointestinal, renal, hepatic, endocrine or cardiovascular system disease, newly treated hypothyroidism, liver function tests greater than three times the upper normal limit, creatinine concentrations greater than 150 mol/L; comorbidity of primary psychiatric (i.e., schizophrenia, major depression onset before the AD onset) or neurological disorders (i.e., stroke, Parkinson disease, seizure disorder, or head injury with loss of consciousness within the past year); known suspected history of alcoholism or drug abuse; computed tomography or magnetic resonance imaging evidence of focal parenchymal abnormalities; Structured Clinical Interview for the DSM-IV (*SCID*-*P*). All of the patients were accurately screened for the onset of depression after the onset of the cognitive symptoms of dementia. Subjects whose depression onset preceded the onset of dementia were excluded.
**Forlenza et al. (2015) [13]**	Case-control study from on-going cohort	-	-	ELISA (DuoSet, 136 R&D Systems, Minneapolis, MN, USA)	-	-	AD patients were under treatment with cholinesterase inhibitors for at least three months at the time of enrolment.	The subjects should not have any evidence of depressive disorder, based on the Hamilton Rating Scale for Depression-21; HDRS-21, Hamilton Rating Scale for Depression-21, APOE genotype.
**Gezen-ak et al. (2013) [29]**	Age-matched case-control study	-	-	ChemiKineTMSandwich ELISA Kit (CYT306, Millipore Corporation, Bil150 lerica, MA, USA)	-, <10%	EOAD: <65 (age at AD onset 50 to 63), LOAD: >65 (age at AD onset 65 to 80), MCI = age at MCI onset 60 to 78	-	Patients with chronic heart disease, inflammatory diseases, autoimmune disease, infectious or psychiatric disease, non-Alzheimer’s dementia, patients taking antibiotics or non-steroidal anti-inflammatory drugs, had significant laboratory abnormalities, did not have erythrocyte sedimentation rates within reference values; -
**Laske et al. (2007) [21]**	Age-matched case-control study	-	-	ELISA kit (R&D Systems GmbH Wiesbaden-Nordenstadt, Germany)	Both <10%	-	-	Excluded those with depressive or psychotic episodes; -
**Laske et al. (2006A) [22]**	Case-control study	-	-	ELISA kit (R&D Systems GmbH Wiesbaden-Nordenstadt, Germany)	Both <10%	-	-	Excluded patients or control subjects with current or a history of depression or psychosis, with neurologic disorders, major physical illness, alcohol or substance abuse, or use of psychoactive medications; NA
**Laske et al. (2006B) [14]**	Age-matched case-control study	-	-	ELISA (R&D Systems GmbH Wiesbaden-Nordenstadt, Germany)	Both <10%	-	-	Excluded those with depressive or psychotic episodes; -
**Laske et al. (2006C) [15]**	Age-matched case-control study	-	-	-	-	-	-	-
**Lee et al. (2009) [16]**	Case-control study	-	-	ELISA kits (Promega, Madison, WI, USA)	-	-	-	With psychotic features or history of depressive episodes according to DSMIV criteria, had clinically significant physical abnormalities based on both physical and laboratory examination, had a history of organic brain abnormality or psychotropic drug misuse; Korean version of the Geriatric Depression Scale (GDS-K). Subjects with total GDS-K scores higher than 20 were considered to have depression.
**Lee et al. (2015) [17]**	Case-control study	-	-	ELISA kits (Promega, Maidison, WI, USA)	-	-	-	Subjects with a history of organic brain abnormalities (e.g., vascular dementia, Parkinson’s disease, etc.); -
**Leyhe et al. (2008) [23]**	Age-matched case-control study	-	-	ELISA kit (R&D Systems GmbH Wiesbaden-Norderstadt, Germany)	Both <10%	-	No known concomitant medication that could interfere with BDNF, specifically no patient received antidepressants, non-steroidal antiphlogistics, or statins.	Excluded those with depressive or psychotic episodes;-
**Platenik et al. (2014) [26]**	Age-matched case-control study	-	-	Human BDNF DuoSet ELISA development kit (cat. no. DY248), Human BDNF Quantikine Immunoassay (cat. no. DBD00)	-	-	AD patients were treated with reversible acetylcholinesterase inhibitors and/or NMDA receptor antagonists, as well as other drugs according to their somatic illnesses. Those with co-morbid depression were treated with antidepressants as well.	Other causes of dementia were excluded, including pseudodementia. Serious somatic disease or chronic somatic pharmacotherapy was not present, and patients were without organic brain disease, without cognitive impairment, and without abuse of psychoactive substances; BMI, platelet concentration, and clinical variables (GDS and MMSE for AD patients).
**O’Bryant et al. (2009) [24]**	Case-control study from a longitudinal research cohort	-	-	Multiplexed immunoassay via human MAP	NA, ≤7%	-	-	NA; NA
**O’Bryant et al. (2010) [25]**	Case-control study from a longitudinal research cohort	-	-	Multiplexed immunoassay via human MAP	NA, ≤7%	-	-	-; GDS and APOE4 status (present versus absent)
**Shimada et al. (2014) [18]**	Case-control study from an observational study	-	-	DuoSet ELISA Development Kit from R&D Systems (Minneapolis, MN, USA). Assays were performed using a specific human BDNF antibody (Minneapolis, MN, USA); no significant cross-reactivity or interference reported in this assay.	3.8, 7.6	-	-	Excluded participants who had missing BDNF data and characteristics, diagnosed neurological disorders, included stroke, Parkinson’s disease, AD, and depression, certified long-term care insurance, or functional decline of activities of daily living (ADL); Walking speed: start and end of 2.4 m walkway, histories of heart diseases and diabetes, smoking status, exercise, frequencies of going outdoors.
**Ventriglia et al. (2013) [27]**	Case-control study	-	-	Human BDNF Quantikine kit (R&D system, Minneapolis, MN, USA)	NA, approximately 8%	-	Most subjects took more than one medication concurrently. Psychotropic medications (neuroleptics, benzodiazepines, antidepressants, mood stabilizers/antiepileptics, L-DOPA, and cholinesterase inhibitors) were recorded and taken into account in the analyses.	-
**Woolley et al. (2012) [11]**	Case-control study; inclusion of multiple neurodegenerative diseases in a single study.	YES; >0.8 power to detect differences in BDNF concentrations in each neurodegenerative disease group when comparing against healthy subjects, assuming the predetermined effect sizes and SDs inthe AD literature.	-	BDNF ELISA kit (R&D Systems, Minneapolis, MN, USA)	<10; 8–14	-	Use of AChEIs or selective serotonin reuptake inhibitors (SSRIs) and/or serotonin and norepinephrine reuptake inhibitors (SNRIs) was analyzed in relation to BDNF concentrations.	-
**Yasutake et al. (2006) [28]**	Age and gender-matched case-control study	-	-	ELISA kit (Quantikine R&D System, Minneapolis, MN, USA)	5, 11.3	-	-	Subjected to a structural interview and physical examination, and those with malignant diseases or severe infections were excluded from all of the study groups.

Abbreviations: ELISA: Enzyme-linked immunosorbent assay; CIBIS+: Clinical Interview Based Impression of Severity with Caregiver Input; NPI: Neuropsychiatric Inventory; AChEI: acetyl-cholinesterase- inhibitor; NMDA: N-Methyl-D-aspartic acid or N-Methyl-D-aspartate; GDS: Geriatric Depression Scale; human MAP: human Multi-Analyte Profile; SSRIs: selective serotonin reuptake inhibitors; SNRIs: serotonin & norepinephrine reuptake inhibitors.DSM-IV: Diagnostic and Statistical Manual of Mental Disorders-5; *SCID*-*P: Structured Clinical Interview for DSM-III-R: Patient Edition; APOE; Apolipoprotein E; EOAD: Early-Onset Alzheimer Disease; LOAD:* Late*-Onset Alzheimer Disease;* “-” represents no relevant information reported.

**Table 3:** Results of meta-regression for serum BDNF levels for patients with AD compared to healthy controls.

Moderators	Number of Studies	β	*z*	*p*
Age	15	0.0548	3.32	<0.001
MMSE Scores	15	0.0518	3.52	<0.001
Sex (% of female)	13	0.0104	1.35	0.1764
Years of Education	7	0.0181	1.06	0.2909
